# Counter-Based Broadcast Scheme Considering Reachability, Network Density, and Energy Efficiency for Wireless Sensor Networks

**DOI:** 10.3390/s18010120

**Published:** 2018-01-04

**Authors:** Ji-Young Jung, Dong-Yoon Seo, Jung-Ryun Lee

**Affiliations:** School of Electrical and Electronics Engineering, Chung-Ang University, 84 Heukseok-ro, Dongjak-gu, Seoul 06974, Korea; jiyoung@cau.ac.kr (J.-Y.J.); dongyoonseo@cau.ac.kr (D.-Y.S.)

**Keywords:** wireless sensor network, counter-based broadcast, reachability, energy efficiency, network density

## Abstract

A wireless sensor network (WSN) is emerging as an innovative method for gathering information that will significantly improve the reliability and efficiency of infrastructure systems. Broadcast is a common method to disseminate information in WSNs. A variety of counter-based broadcast schemes have been proposed to mitigate the broadcast-storm problems, using the count threshold value and a random access delay. However, because of the limited propagation of the broadcast-message, there exists a trade-off in a sense that redundant retransmissions of the broadcast-message become low and energy efficiency of a node is enhanced, but reachability become low. Therefore, it is necessary to study an efficient counter-based broadcast scheme that can dynamically adjust the random access delay and count threshold value to ensure high reachability, low redundant of broadcast-messages, and low energy consumption of nodes. Thus, in this paper, we first measure the additional coverage provided by a node that receives the same broadcast-message from two neighbor nodes, in order to achieve high reachability with low redundant retransmissions of broadcast-messages. Second, we propose a new counter-based broadcast scheme considering the size of the additional coverage area, distance between the node and the broadcasting node, remaining battery of the node, and variations of the node density. Finally, we evaluate performance of the proposed scheme compared with the existing counter-based broadcast schemes. Simulation results show that the proposed scheme outperforms the existing schemes in terms of saved rebroadcasts, reachability, and total energy consumption.

## 1. Introduction

A wireless sensor network (WSN) is a network formed by a large number of sensor nodes, who detect the physical phenomena of the surrounding environments and report the sensed data to a sink node through multi-hop links. WSNs are regarded as an innovative method for gathering information that will significantly improve the reliability and efficiency of infrastructure systems such as smart grid, smart homes, smart industrial automation, etc. Broadcasting is a common method for disseminating information in a WSN, when a node intends to share its data efficiently among each other. When the source node transmits a broadcast-message, the receiving nodes retransmit this message to all other nodes in their transmission range, and, thus, information exchange is possible between nodes in a multi-hop distance [1,2]. Flooding, in which each node retransmits every uniquely received broadcast-message exactly once, is the simplest broadcast scheme guaranteeing high reachability. However, it can result in a large number of redundant transmissions of the same broadcast-message, which can cause high channel contention and collisions. This phenomenon is referred to as the broadcast-storm problem [3,4]. In addition, in a WSN, nodes act as both source/destination nodes as well as relay nodes, simultaneously. Thus, nodes may waste their energy for relaying data that just passes by the node [5,6]. Because a node with a discharged battery resulting from such high energy consumption cannot act as a relay node anymore, it becomes a major cause of the deterioration in network performance. Thus, the energy efficiency of a node is an important performance metric to be considered carefully in the design of a broadcasting scheme for WSNs.

To mitigate the broadcast-storm problem, counter-based broadcast schemes have been proposed. In counter-based broadcast schemes, a node receiving a broadcast-message sets the count value (*c*) and random access delay (RAD) to keep track of the number of duplicate broadcast-messages received. Next, the node counts the number of duplicate broadcast-messages received during RAD. As soon as the RAD timer expires, the node compares the count value with a fixed count threshold value ($C_{th}$). If $c\leq C_{th}$, the node transmits the broadcast-message; otherwise, transmission of the broadcast-message is inhibited. Thus, the counter-based broadcast scheme can mitigate the problems of collision and redundancy in broadcast-message retransmissions in blind flooding [7,8]. The performance of the counter-based broadcast scheme is greatly affected by the count threshold value and random access delay. For example, when the count threshold value is set too low or a too short random access delay is used, the possibility that a node relays broadcast-messages decreases. Then, the propagation of the broadcast-message is limited, and, therefore, the reachability deteriorates. Nevertheless, in this case, the redundancy in the retransmission of the broadcast-message and the energy consumption of the nodes decrease, owing to the small number of the retransmissions of the broadcast-message. It means that the use of RAD and $C_{th}$ in counter-based broadcast schemes involves a trade-off in a sense that the use of a short RAD and small $C_{th}$ results in low reachability, reduced redundancy in the retransmission of broadcast-messages, and high energy efficiencies for the nodes in the network. Node density is another important factor that affects the performance of the counter-based broadcasting schemes. For a given RAD and $C_{th}$, nodes in dense areas have higher probabilities of transmitting the broadcast-messages than those in sparse areas. Therefore, high reachability is achieved and the energy efficiency is improved; however, the redundancy via the retransmission of broadcast-messages increases when the node density is high. Thus, an efficient counter-based broadcast scheme should dynamically adjust the random access delay and count threshold value to ensure high reachability, low number of redundant retransmissions of broadcast-messages, and high energy efficiency of nodes, according to the variations in the node density.

For this purpose, in this paper, we first measure the degree of reachability more accurately than the previous distance-aware counter-based broadcasting scheme, by using not only the distance between a node and the broadcasting node but also the additional coverage area provided by a node that receives the same broadcast-message from two neighbor nodes. Second, we propose an efficient counter-based broadcast scheme, which determines the RAD and $C_{th}$ such that high reachability, high energy efficiency, and low redundancy in broadcast-message transmission are achieved. The rest of this paper is organized as follows. Section 2 describes the related works on various counter-based broadcast schemes. In Section 3, we propose an efficient counter-based broadcast scheme, which considers the reachability, node density, and energy efficiency, simultaneously. In Section 4, we provide simulation results and discuss the performances of the proposed scheme. Finally, conclusions are given in Section 5.

## 2. Related Works

In this section, we introduce three representative counter-based broadcast schemes: (1) distance-aware counter-based broadcast (DCB), (2) neighborhood-aware counter-based broadcast (NCB), and (3) battery-aware counter-based broadcast (BCB).

### 2.1. Distance-Aware Counter-Based Broadcast (DCB) Scheme

**Figure 1:**
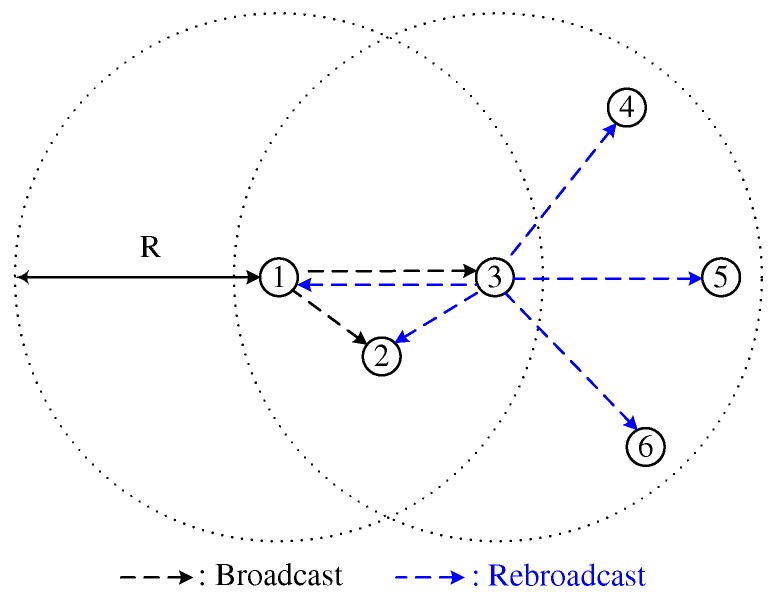
Distance-aware counter-based broadcast scheme.

Sun et al. [9] proposed a DCB scheme in which a node receiving a broadcast-message determined an RAD based on the distance between itself and the broadcasting node. Let $T_{max}$ be the maximum random access delay, *R* be the maximum transmission range of the node, and *D* be the distance between the broadcasting and receiving nodes. Then, in order for a node that is far from (close to) the broadcasting node to use a short (long) RAD, the RAD is defined as follows:

(1) $$RAD=rand\left[0,1\right]\times T_{max}\times \frac{\left(R^{2}-D^{2}\right)}{R^{2}},$$

where rand[0,1] is a uniformly distributed random variable between 0 and 1. In DCB, $C_{th}$ is defined as a fixed value. Because the coverage area of a node close to the broadcasting node is narrower than that of a node far from the broadcasting node, as shown in Figure 1, DCB mitigates the broadcast-storm problem by prohibiting the broadcast from node 2, thus providing high reachability. In DCB, distance between the broadcasting and receiving nodes can be obtained from a global positioning system (GPS) device. However, when GPS information is unavailable or unreliable, a dissimilarity-based method can be applied, in which the number of neighbors shared by two nodes is used as a performance metric to estimate the relative distance between them [10].

### 2.2. Neighborhood-Aware Counter-Based Broadcast (NCB) Scheme

Humoud et al. [11] proposed an NCB scheme in which the RAD was determined based on the number of neighbor nodes. Let RF be a random factor. RF is set as one of two values: $RF_{1}$ or $RF_{2}$, where $RF_{1}$ is less than $RF_{2}$. When a node receives a broadcast-message, it checks the number of its neighbor nodes, *n*, against the average number of neighbor nodes, $n_{avg}$. If $n<n_{avg}$ ($n\geq n_{avg}$), the network is considered sparse (dense), and RF is set to $RF_{1}$ ($RF_{2}$). Thus, a node with a large (small) number of neighbor nodes determines a short (long) RAD as follows:

(2) $$RAD=\frac{rand[0,1]}{RF}.$$

**Figure 2:**
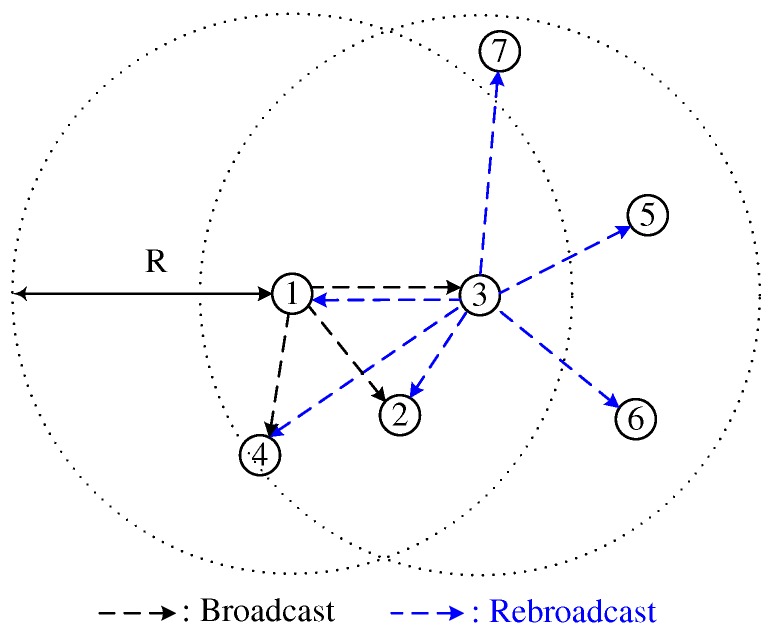
Neighborhood-aware counter-based broadcast scheme.

In addition, a node with a large (small) number of neighbor nodes determines a small (large) $C_{th}$. In Figure 2, the number of neighbor nodes of node 3 is greater than that of nodes 2 and 4. In this case, NCB mitigates the broadcast-storm problem by prohibiting the broadcasts from nodes 2 and 4, and can guarantee high reachability by rebroadcasting the broadcast-messages of node 3 to more neighbor nodes.

### 2.3. Battery-Aware Counter-Based Broadcast (BCB) Scheme

**Figure 3:**
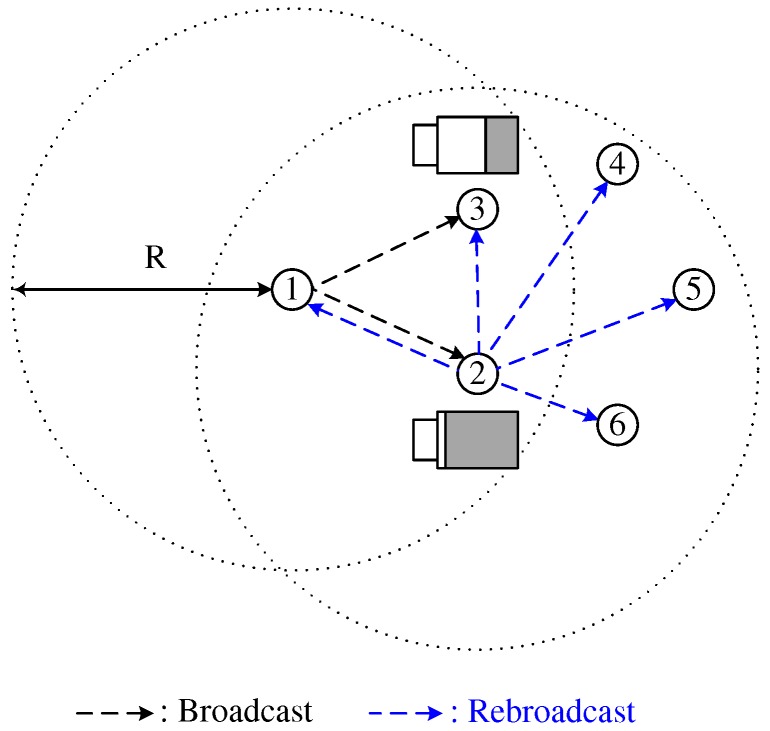
Battery-aware counter-based broadcast scheme.

Utsu et al. [12] proposed a battery-aware counter-based broadcast (BCB) scheme in which a node receiving a broadcast-message determined both $C_{th}$ and RAD, based on the remaining battery level. Let $C_{max}$, $B_{R}$, and $B_{max}$ be the maximum count threshold value, the remaining battery of a receiving node, and the maximum battery charge, respectively. Then, in order to make a node with a high (low) battery level employ a large (small) $C_{th}$, $C_{th}$ is defined as follows:

(3) $$C_{th}=ceil\left(C_{max}\times \frac{B_{R}}{B_{max}}\right),$$

where ceil(x) is the ceiling function, which provides the smallest integer greater than or equal to *x*. On the other hand, RAD is defined by

(4) $$RAD=rand\left[0,1\right]\times 2\left(C_{max}-C_{th}\right),$$

which is designed for a node with a high (low) battery level to opportunistically employ a short (long) RAD. In Figure 3, the remaining battery level of node 2 is higher than that of node 3. In this case, BCB mitigates the broadcast-storm problem while improving the energy efficiency, by prohibiting the broadcast of node 3 with a low battery level.

As mentioned previously, the DCB and NCB were proposed to achieve high reachability; on the other hand, the BCB was proposed to reduce the node’s energy consumption. Here, we notice that there is a trade-off between the reachability and the energy consumption of nodes in a wireless ad hoc network. If a specific node in DCB is far from the broadcasting node, it transmits broadcast-messages repeatedly, which causes high energy consumption for the node, and network-partition problems may occur. Similar arguments can be applied to a node that has a large number of neighboring nodes in NCB. In contrast, when focusing on reducing the battery consumption of a node, if a specific node in BCB has a high battery level, but is close to the broadcasting node or has a small number of neighboring nodes, it transmits the broadcast-message repeatedly although it is difficult to guarantee high reachability.

## 3. Proposed Method

**Figure 4:**
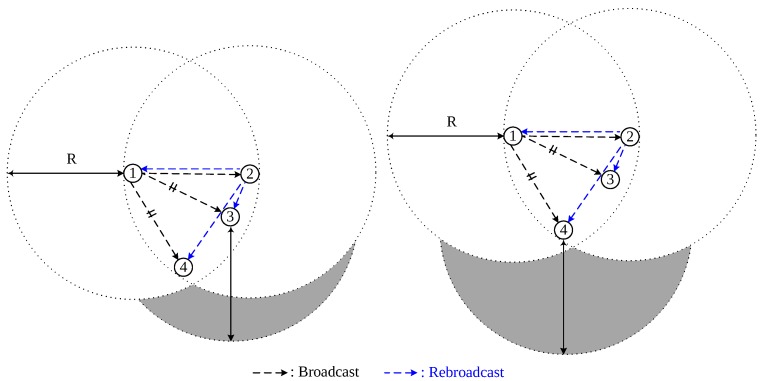
Different additional coverage areas of node 3 and node 4.

In Figure 4, nodes 3 and 4 are equidistant from node 1 and receive the same broadcast-messages from both nodes 1 and 2. Here, it is noticed that, although nodes 3 and 4 are equally far from node 1, the additional coverage of node 4 is wider than that of node 3 because of the geographical locations of the nodes. This explains the necessity to calculate the exact additional coverage areas of the nodes that receive the same broadcast-message from two different nodes, so that nodes with the largest additional coverage areas are more likely to be selected for rebroadcasting the packets, thereby achieving high reachability.

### 3.1. Calculation of Additional Coverage Area

**Figure 5:**
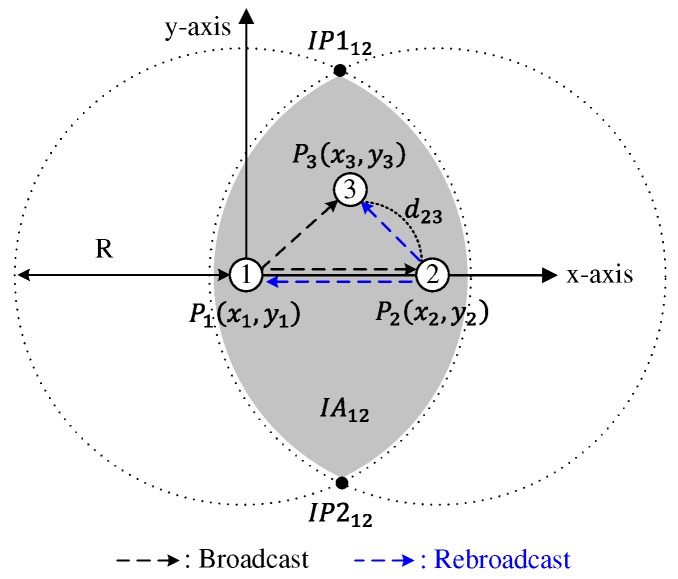
Nodes in the shadowed area receive the same broadcast-message from nodes 1 and 2.

Suppose that node 1 sends a broadcast-message and node 2 rebroadcasts this message. Then, node 3 in the intersection area of the coverage areas of nodes 1 and 2 (shadowed area in Figure 5) receives the same broadcast-message from both nodes 1 and 2. It is assumed that each node is equipped with a GPS, and, thus, the distance between two nodes is recognized by the receiving node via the broadcast-message containing the GPS information of the sender [13]. Let *R* and $d_{ij}$ be the transmission range of a node and the distance between nodes *i* and *j*, respectively. Because node 3 receives the broadcast-messages delivered through node 1 and node 2, it can recognize the distances $d_{12},d_{23}$, and $d_{13}$. Let the point of node *i* in the two-dimensional Euclidean space be $P_{i}$ and the coordinates of the node *i* be ($x_{i}$, $y_{i}$). Without loss of generality, we assume that node 1 is located at the origin point and that node 2 is on the right-hand side of the *x*-axis, as shown in Figure 5. That is,

(5) $$\begin{array}{c} P_{1}=\left(0,0\right),\;P_{2}=\left(d_{12},0\right). \end{array}$$

Using $d_{12}$, $d_{13}$, and $d_{23}$, $P_{3}$ is expressed by

(6) $$\begin{array}{c} P_{3}=(\frac{d_{13}^{2}+d_{12}^{2}-d_{23}^{2}}{2d_{12}},\frac{\sqrt{1-(d_{13}^{2}+d_{12}^{2}-d_{23}^{2})}}{2d_{12}}). \end{array}$$

When the distance between two nodes is less than the transmission range *R*, there are two intersection points between two transmission ranges. Let the two intersection points between the transmission ranges of nodes *i* and *j* and the intersection area of the coverage areas of nodes *i* and *j* be $IP1_{ij}$, $IP2_{ij}$, and $IA_{ij}$, respectively. Then, assuming that ${IP1}_{ij}$ is located above ${IP2}_{ij}$, as shown in Figure 5, the coordinates of the points ${IP1}_{12}$ and ${IP2}_{12}$ are calculated by

(7) $$\begin{array}{c} {IP1}_{12}=(\frac{d_{12}}{2},\sqrt{R^{2}-(\frac{d_{12}}{2}{)}^{2}}), \end{array}$$

(8) $$\begin{array}{c} {IP2}_{12}=(\frac{d_{12}}{2},-\sqrt{R^{2}-(\frac{d_{12}}{2}{)}^{2}}). \end{array}$$

As shown in Figure , the shape of the additional coverage area of node 3 is classified into three cases according to the following conditions for node 3:

1. $|\vec{P_{3}{IP1}_{12}}|<R$ and $|\vec{P_{3}{IP2}_{12}}|<R$,
2. $|\vec{P_{3}{IP1}_{12}}|>R$ and $|\vec{P_{3}{IP2}_{12}}|>R$,
3. $\left(|\vec{P_{3}{IP1}_{12}}|-R\right)\cdot \left(|\vec{P_{3}{IP2}_{12}}|-R\right)<0$.

**Figure 7:**
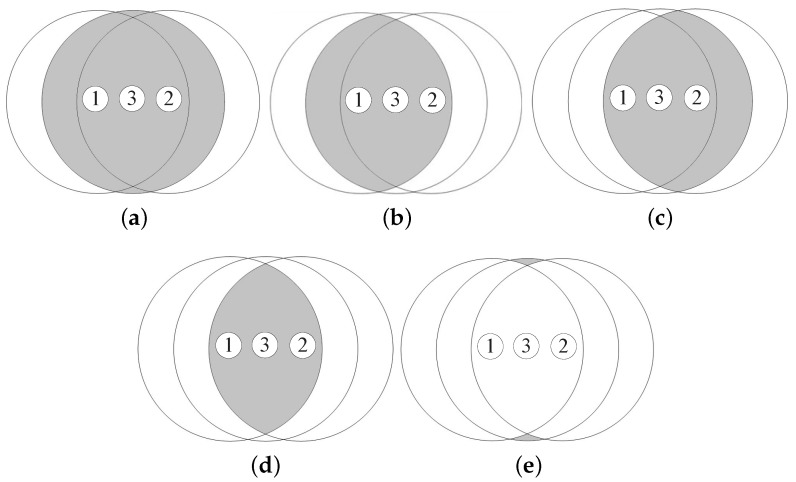
The additional coverage area of node 3 in case 1. (**a**) $TA_{3}$; (**b**) $IA_{13}$; (**c**) $IA_{23}$; (**d**) $IA_{12}$; (**e**) $AC_{1}$.

Here, we define the transmission area of node *i* as ${TA}_{i}$ and the additional coverage area of node 3 in case *i* as ${AC}_{i}$. Then, from Figure 7, ${AC}_{1}$ is given by

(9) $${AC}_{1}={TA}_{3}-{IA}_{13}-{IA}_{23}+IA_{12}.$$

On the other hand, ${TA}_{3}$, ${IA}_{13}$, ${IA}_{23}$, and ${IA}_{12}$ is calculated as follows:

(10) $${TA}_{3}=\pi R^{2},$$

(11) $$\begin{array}{c} IA_{13}=4\int_{\frac{d_{13}}{2}}^{R}\sqrt{R^{2}-x^{2}}dx=2R^{2}\cos^{-1}(\frac{d_{13}}{2R})-\frac{d_{13}\sqrt{4R^{2}-d_{13}^{2}}}{2}, \end{array}$$

(12) $$\begin{array}{c} IA_{23}=4\int_{\frac{d_{23}}{2}}^{R}\sqrt{R^{2}-x^{2}}dx=2R^{2}\cos^{-1}(\frac{d_{23}}{2R})-\frac{d_{23}\sqrt{4R^{2}-d_{23}^{2}}}{2}, \end{array}$$

(13) $$\begin{array}{c} IA_{12}=4\int_{\frac{d_{12}}{2}}^{R}\sqrt{R^{2}-x^{2}}dx=2R^{2}\cos^{-1}(\frac{d_{12}}{2R})-\frac{d_{12}\sqrt{4R^{2}-d_{12}^{2}}}{2}. \end{array}$$

Combining Equations (9)–(13) results in the expression of $AC_{1}$ as a function of the distance between the three nodes, which is given by

(14) $$\begin{array}{c} AC_{1}=\pi R^{2}-2R^{2}\{\cos^{-1}(\frac{d_{13}}{2R})+\cos^{-1}(\frac{d_{23}}{2R})-\cos^{-1}(\frac{d_{12}}{2R})\}\\ +\frac{d_{13}\sqrt{4R^{2}-d_{13}^{2}}}{2}+\frac{d_{23}\sqrt{4R^{2}-d_{23}^{2}}}{2}-\frac{d_{12}\sqrt{4R^{2}-d_{12}^{2}}}{2}. \end{array}$$

**Figure 8:**
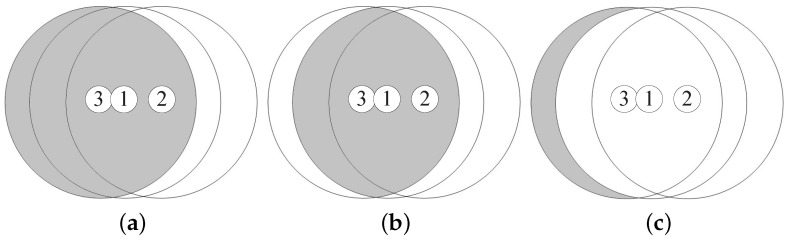
The additional coverage area of node 3 in case 2. (**a**) $TA_{3}$; (**b**) $IA_{13}$; (**c**) $AC_{2}$.

From Figure 8, the additional coverage area of node 3 is calculated by

(15) $$AC_{2}=\left\{\begin{array}{ccc} TA_{3}-IA_{13}, & \mathrm{if} & \left|\vec{P_{3}P_{1}}\right|<\left|\vec{P_{3}P_{2}}\right|\\ TA_{3}-IA_{23}, & \mathrm{if} & \left|\vec{P_{3}P_{1}}\right|>\left|\vec{P_{3}P_{2}}\right| \end{array}\right..$$

Combining Equations (10)–(12) and (15) results in the expression of $AC_{2}$ as a function of the distance between the three nodes, which is given by

(16) $$AC_{2}=\left\{\begin{array}{ccc} \pi R^{2}-2R^{2}\cos^{-1}\left(\frac{d_{13}}{2R}\right)+\frac{d_{13}\sqrt{4R^{2}-d_{13}^{2}}}{2}, & \mathrm{if} & \left|\vec{P_{3}P_{1}}\right|<\left|\vec{P_{3}P_{2}}\right|,\\ \pi R^{2}-2R^{2}\cos^{-1}\left(\frac{d_{23}}{2R}\right)+\frac{d_{23}\sqrt{4R^{2}-d_{23}^{2}}}{2}, & \mathrm{if} & \left|\vec{P_{3}P_{1}}\right|>\left|\vec{P_{3}P_{2}}\right|. \end{array}\right.$$

**Figure 9:**
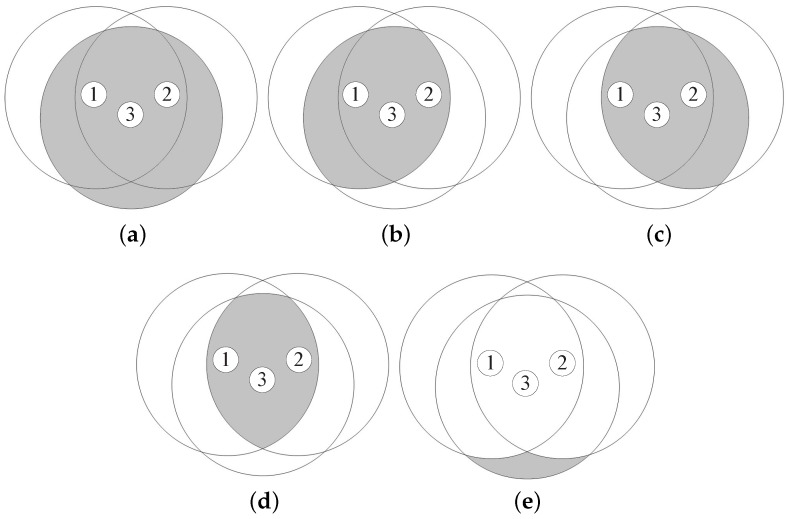
The additional coverage area of node 3 in case 3. (**a**) $TA_{3}$; (**b**) $IA_{13}$; (**c**) $IA_{23}$; (**d**) $IA_{123}$; (**e**) $AC_{3}$.

Let the intersection area of the coverages of nodes *i*, *j*, and *k* be $IA_{ijk}$. Then, from Figure 9, we can obtain the additional coverage area of node 3 in case 3, which is given by

(17) $$AC_{3}=TA_{3}-{IA}_{13}-IA_{23}+IA_{123}.$$

**Figure 10:**
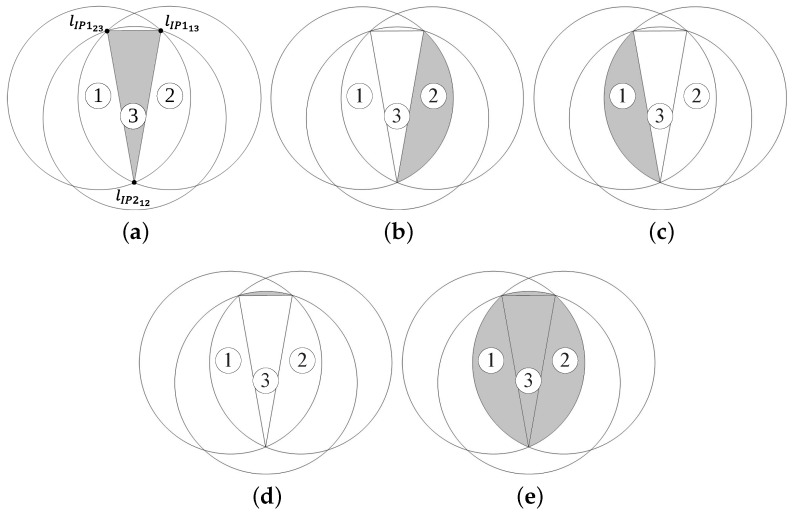
An intersection area within the transmission range of three nodes. (**a**) $S_{1}$; (**b**) $S_{2}$; (**c**) $S_{3}$; (**d**) $S_{4}$; (**e**) $IA_{123}$.

From Figure 10, $IA_{123}$ is calculated as follows:

(18) $$IA_{123}=S_{1}+S_{2}+S_{3}+S_{4},$$

where $S_{i}$ is shown in Figure 10a–d. In case $P_{3}$ is located below the *x*-axis, the coordinates of the points $IP1_{13}$ and $IP1_{23}$ are calculated by

(19) $$\begin{array}{cc} IP1_{13} & =\left(\frac{-b_{1}+\sqrt{b_{1}^{2}-4a_{1}c_{1}}}{2a_{1}},\frac{-e_{1}+\sqrt{e_{1}^{2}-4d_{1}f_{1}}}{2d_{1}}\right),\\ \mathrm{where} & a_{1}=1+\frac{x_{3}^{2}}{y_{3}^{2}},\;b_{1}=-x_{3}-\frac{x_{3}^{3}}{y_{3}^{2}},\;c_{1}=\frac{x_{3}^{4}}{4y_{3}^{2}}+\frac{x_{3}^{2}}{2}+\frac{y_{3}^{2}}{4}-R^{2},\\ & d_{1}=1+\frac{y_{3}^{2}}{x_{3}^{2}},\;e_{1}=-y_{3}-\frac{y_{3}^{3}}{x_{3}^{2}},\;f_{1}=\frac{y_{3}^{4}}{4x_{3}^{2}}+\frac{y_{3}^{2}}{2}+\frac{x_{3}^{2}}{4}-R^{2}, \end{array}$$

and

(20) $$\begin{array}{cc} IP1_{23} & =\left(\frac{-b_{2}-\sqrt{b_{2}^{2}-4a_{2}c_{2}}}{2a_{2}},\frac{-e_{2}+\sqrt{e_{2}^{2}-4d_{2}f_{2}}}{2d_{2}}\right),\\ \mathrm{where} & a_{2}=1+\frac{{\left(x_{3}-x_{2}\right)}^{2}}{y_{3}^{2}},\;b_{2}=-x_{3}-x_{2}-\frac{\left(x_{2}+x_{3}\right){\left(x_{3}-x_{2}\right)}^{2}}{y_{3}^{2}},\\ & c_{2}=\frac{{\left(x_{3}-x_{2}\right)}^{2}{\left(x_{3}+x_{2}\right)}^{2}}{4y_{3}^{2}}+\frac{\left(x_{3}^{2}-x_{2}^{2}\right)}{2}+\frac{y_{3}^{2}}{4}+x_{2}^{2}-R^{2},\\ & d_{2}=1+\frac{y_{3}^{2}}{{\left(x_{3}-x_{2}\right)}^{2}},\;e_{2}=-y_{3}-\frac{y_{3}^{3}}{{\left(x_{3}-x_{2}\right)}^{2}},\\ & f_{2}=\frac{y_{3}^{4}}{4{\left(x_{3}-x_{2}\right)}^{2}}+\frac{y_{3}^{2}}{2}+\frac{{\left(x_{3}-x_{2}\right)}^{2}}{4}-R^{2}, \end{array}$$

respectively. From Heron’s formula, $S_{1}$ is calculated as follows:

(21) $$\begin{array}{c} S_{1}=\frac{1}{4}\sqrt{\left(d_{a}+d_{b}+d_{c}\right)\left(d_{a}+d_{b}-d_{c}\right)\left(d_{b}+d_{c}-d_{a}\right)\left(d_{c}+d_{a}-d_{b}\right)},\\ \mathrm{where}\;d_{a}=|\vec{IP2_{12}IP1_{13}}|,d_{b}=|\vec{IP2_{12}IP1_{23}}|,d_{c}=\left|\vec{IP1_{13}IP1_{23}}\right|. \end{array}$$

On the other hand, $S_{2}$, $S_{3}$, and $S_{4}$ are calculated as follows:

(22) $$S_{2}=\frac{1}{2}R^{2}\left(\theta_{1}-\sin\theta_{1}\right),$$

(23) $$S_{3}=\frac{1}{2}R^{2}\left(\theta_{2}-\sin\theta_{2}\right),$$

(24) $$S_{4}=\frac{1}{2}R^{2}\left(\theta_{3}-\sin\theta_{3}\right),$$

where $\theta_{1}=\cos^{-1}(\frac{\vec{P_{1}IP2_{12}}\cdot \vec{P_{1}IP1_{23}}}{R^{2}})$, $\theta_{2}=\cos^{-1}(\frac{\vec{P_{2}IP1_{13}}\cdot \vec{P_{2}IP2_{12}}}{R^{2}})$, and $\theta_{3}=\cos^{-1}(\frac{\vec{P_{3}IP1_{13}}\cdot \vec{P_{3}IP1_{23}}}{R^{2}})$. Combining Equations (18), (21)–(24) results in the expression of $IA_{123}$ as

(25) $$\begin{array}{c} IA_{123}=\frac{1}{4}\sqrt{\left(d_{a}+d_{b}+d_{c}\right)\left(d_{a}+d_{b}-d_{c}\right)\left(d_{b}+d_{c}-d_{a}\right)\left(d_{c}+d_{a}-d_{b}\right)}\\ +\frac{1}{2}R^{2}\left(\theta_{1}+\theta_{2}+\theta_{3}-\sin\theta_{1}-\sin\theta_{2}-\sin\theta_{3}\right). \end{array}$$

Thus, combining Equations (10)–(12), (17), and (25) results in the expression of $AC_{3}$ as

(26) $$\begin{array}{c} AC_{3}=R^{2}(\pi -2\cos^{-1}(\frac{d_{13}}{2R})-2\cos^{-1}(\frac{d_{23}}{2R}))\\ \;+\frac{R^{2}}{2}\left(\theta_{1}+\theta_{2}+\theta_{3}-\sin\theta_{1}-\sin\theta_{2}-\sin\theta_{3}\right)\\ \;+\frac{d_{13}\sqrt{4R^{2}-d_{13}^{2}}}{2}+\frac{d_{23}\sqrt{4R^{2}-d_{23}^{2}}}{2}\\ \;+\frac{1}{4}\sqrt{\left(d_{a}+d_{b}+d_{c}\right)\left(d_{a}+d_{b}-d_{c}\right)\left(d_{b}+d_{c}-d_{a}\right)\left(d_{c}+d_{a}-d_{b}\right)}. \end{array}$$

In case $P_{3}$ is located above the *x*-axis, we can get the $AC_{3}$ from Equation (26) by substituting $IP1_{12}$, $IP2_{13}$, $IP2_{23}$ for $IP2_{12}$, $IP1_{13}$, $IP1_{23}$, respectively. The coordinates of the points $IP2_{13}$ and $IP2_{23}$ are calculated by

(27) $$\begin{array}{cc} IP2_{13} & =\left(\frac{-b_{1}+\sqrt{b_{1}^{2}-4a_{1}c_{1}}}{2a_{1}},\frac{-e_{1}-\sqrt{e_{1}^{2}-4d_{1}f_{1}}}{2d_{1}}\right),\\ \mathrm{where} & a_{1}=1+\frac{x_{3}^{2}}{y_{3}^{2}},\;b_{1}=-x_{3}-\frac{x_{3}^{3}}{y_{3}^{2}},\;c_{1}=\frac{x_{3}^{4}}{4y_{3}^{2}}+\frac{x_{3}^{2}}{2}+\frac{y_{3}^{2}}{4}-R^{2},\\ & d_{1}=1+\frac{y_{3}^{2}}{x_{3}^{2}},\;e_{1}=-y_{3}-\frac{y_{3}^{3}}{x_{3}^{2}},\;f_{1}=\frac{y_{3}^{4}}{4x_{3}^{2}}+\frac{y_{3}^{2}}{2}+\frac{x_{3}^{2}}{4}-R^{2}, \end{array}$$

and

(28) $$\begin{array}{cc} IP2_{23} & =\left(\frac{-b_{2}-\sqrt{b_{2}^{2}-4a_{2}c_{2}}}{2a_{2}},\frac{-e_{2}-\sqrt{e_{2}^{2}-4d_{2}f_{2}}}{2d_{2}}\right),\\ \mathrm{where} & a_{2}=1+\frac{{\left(x_{3}-x_{2}\right)}^{2}}{y_{3}^{2}},\;b_{2}=-x_{3}-x_{2}-\frac{\left(x_{2}+x_{3}\right){\left(x_{3}-x_{2}\right)}^{2}}{y_{3}^{2}},\\ & c_{2}=\frac{{\left(x_{3}-x_{2}\right)}^{2}{\left(x_{3}+x_{2}\right)}^{2}}{4y_{3}^{2}}+\frac{\left(x_{3}^{2}-x_{2}^{2}\right)}{2}+\frac{y_{3}^{2}}{4}+x_{2}^{2}-R^{2},\\ & d_{2}=1+\frac{y_{3}^{2}}{{\left(x_{3}-x_{2}\right)}^{2}},\;e=-y_{3}-\frac{y_{3}^{3}}{{\left(x_{3}-x_{2}\right)}^{2}},\\ & f_{2}=\frac{y_{3}^{4}}{4{\left(x_{3}-x_{2}\right)}^{2}}+\frac{y_{3}^{2}}{2}+\frac{{\left(x_{3}-x_{2}\right)}^{2}}{4}-R^{2}, \end{array}$$

respectively.

### 3.2. Proposed Algorithm to Determine a Random Access Delay

As RAD gets longer, the number of nodes transmitting the same broadcast-message increases, and thus, the reachability improves. However, the energy efficiency decreases because the number of transmitted broadcast-messages increases the battery consumptions of the nodes in the network. Thus, it is important to determine the RAD considering the trade-off between the reachability and the energy efficiency of nodes in the network.

**Figure 11:**
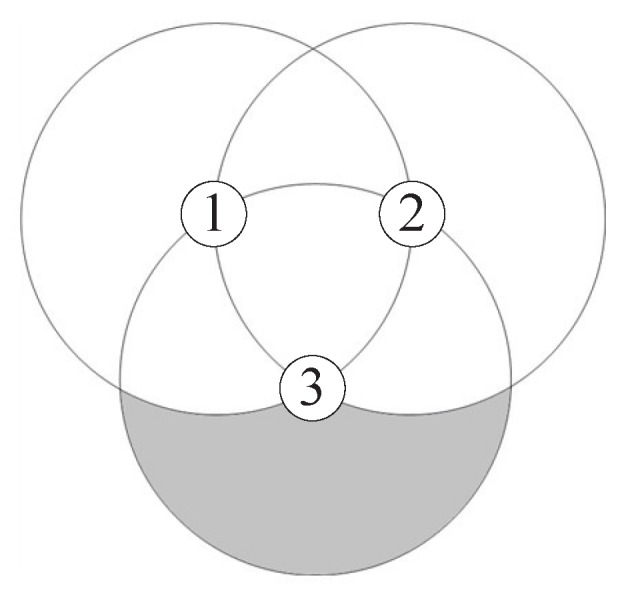
The maximum additional coverage area achieved by node 3

Suppose that node 3 is located in the overlapped coverage area of two nodes 1 and 2. $AC_{max}$, the maximum additional coverage area of node 3, is obtained when the distances between the three nodes are equal to *R*, that is, $d_{12}=d_{23}=d_{13}=R$, as shown in Figure 11. Thus, $AC_{max}$ is calculated by

(29) $$AC_{max}=\frac{\pi R^{2}}{6}+\frac{\sqrt{3}R^{2}}{2}.$$

In order to allow a node with wider additional coverage area to transmit a broadcast-message earlier than the other nodes, we define the random access delay $RAD_{C}$ considering the additional coverage area of a node as

(30) $$RAD_{C}=rand\left[0,1\right]\times (1-\frac{AC_{k}}{AC_{max}}),$$

where $AC_{k}$ is the additional coverage area of a node *k* and rand[0,1] is a uniformly distributed random variable between 0 and 1.

For a node that is far from (close to) the broadcasting node to use a short (long) random access delay, we define the random access delay $RAD_{D}$ considering the distance between the node and the broadcasting node as

(31) $$RAD_{D}=rand\left[0,1\right]\times (1-\frac{d_{tk}}{R}),$$

where $d_{tk}$ is the distance between a node *k* and the broadcasting node.

Let $E_{k}$, $E_{max}$, and $RAD_{B}$ be the remaining battery of node *k*, maximum battery charge, and random access delay considering the remaining battery level of a node. In order for a node that has high (low) remaining battery to use a short (long) random access delay, we define $RAD_{B}$ as

(32) $$RAD_{B}=rand\left[0,1\right]\times (1-\frac{E_{k}}{E_{max}}).$$

Finally, we define the random access delay of a node *k* in the network as

(33) $$RAD=\alpha RAD_{B}+\beta RAD_{C}+\gamma RAD_{D},$$

where α∈[0,1], β∈[0,1], and γ∈[0,1] are weighted parameters determined by the density of neighbor nodes, as follows:

(34) $$\begin{array}{c} \alpha =\frac{N_{k_{neighbor}}}{N_{max}}, \end{array}$$

(35) $$\begin{array}{c} \beta =\frac{1}{2}\left(1-\frac{N_{k_{neighbor}}}{N_{max}}\right), \end{array}$$

(36) $$\begin{array}{c} \gamma =1-\alpha -\beta , \end{array}$$

where $N_{k_{neighbor}}$ and $N_{max}$ are the number of neighbor nodes of node *k* and the total number of nodes in the network, respectively.

When a node is located in a dense area, α increases, and a node with a high remaining amount of battery is therefore more likely to be selected for the rebroadcasting, thereby achieving high energy efficiency. On the other hand, when a node is located in a sparse area, β and γ increase, and a node that is far from the broadcasting node or a node that achieves a wider additional coverage area is more likely to be selected for the rebroadcasting. Thus, high reachability and lower redundancy of broadcast-messages can be achieved.

### 3.3. Proposed Algorithm to Determine a Fixed Count Threshold Value

When large $C_{th}$ is employed, the number of nodes transmitting the same broadcast-message decreases. Therefore, energy efficiency is improved, but reachability deteriorates. Thus, it is important to determine $C_{th}$ considering the trade-off between reachability and energy efficiency of nodes in the network.

Let $C_{min}$, $C_{max}$, and $N_{avg}$ be the minimum and maximum count threshold values, and the average number of neighbor nodes, respectively. Then, the count threshold of node *k* is defined by

(37) $$C_{th}^{k}=C_{min}+c_{k}\times \left(C_{max}-C_{min}\right),$$

where $c_{k}$ is defined as

(38) $$c_{k}=\left\{\begin{array}{ccc} \frac{E_{k}}{E_{max}}, & \mathrm{if} & N_{k_{neighbor}}\geq N_{avg},\\ 1-\frac{N_{k_{neighbor}}}{N_{max}}, & \mathrm{if} & N_{k_{neighbor}}<N_{avg}. \end{array}\right.$$

From Equations (37) and (38), the count threshold value of a node in a sparse environment ($N_{k_{neighbor}}<N_{avg}$) is determined based on the number of neighbor nodes and has a large value (close to 1) in order to improve reachability and avoid the network-partitioning problem. On the other hand, when a node is in a dense environment ($N_{k_{neighbor}}\geq N_{avg}$), its count threshold value is set according to the remaining battery of the node with the purpose of reducing the battery consumption of the node and redundancy of the broadcast-messages.

## 4. Performance Evaluation

### 4.1. Simulation Environment

The performance of the proposed counter-based broadcast scheme is compared against those of the existing counter-based broadcast schemes: the simplest counter-based broadcast scheme (SCB), in which the node transmits the broadcast-message when $c<C_{th}$, and the DCB, NCB, and BCB introduced in Section 2. Table 1 shows the parameters used in the simulation runs. We assume a static network topology, as can be verified in most of the applications of wireless sensor networks [14,15,16]. In simulation, the source nodes of broadcasting packets are selected using partition degree (PD) and average hop to reach the reachable nodes (AHRN) [17] to guarantee a fair comparison. To observe the performance variations according to node density, we vary the variance of the number of neighbor nodes. It is noticed that the variance of the number of neighbor nodes become small when the nodes are evenly distributed, whereas it is large when the nodes are clustered in a certain area.

For the performance metrics, we employ saved rebroadcast (SRB), reachability (RE), and total energy consumption (TEC). SRB is defined as $1-(N_{TB}/N_{RB})$, where $N_{TB}$ is the number of nodes that actually transmitted the broadcast-message, and $N_{RB}$ is the number of nodes receiving the broadcast-message. RE is defined as $N_{RB}/N_{R}$, where $N_{R}$ is the number of nodes that are reachable, directly or indirectly, from the source node. TEC is defined as the total energy consumption of all nodes in the network, until the simulation run is over. The transmission power, $P_{TX}\left(uW\times sec\right)$, and reception power, $P_{RX}\left(uW\times sec\right)$, for a broadcast message are defined as follows [12]:

(39) $$P_{TX}=2\times broadcastmessagelength\left[bytes\right]+270,$$

(40) $$P_{RX}=0.5\times broadcastmessagelength\left[bytes\right]+60.$$

Monte Carlo simulations for 1000 trials are performed using the C language to evaluate the performance of the proposed and comparison algorithms.

### 4.2. Simulation Results

**Figure 12:**
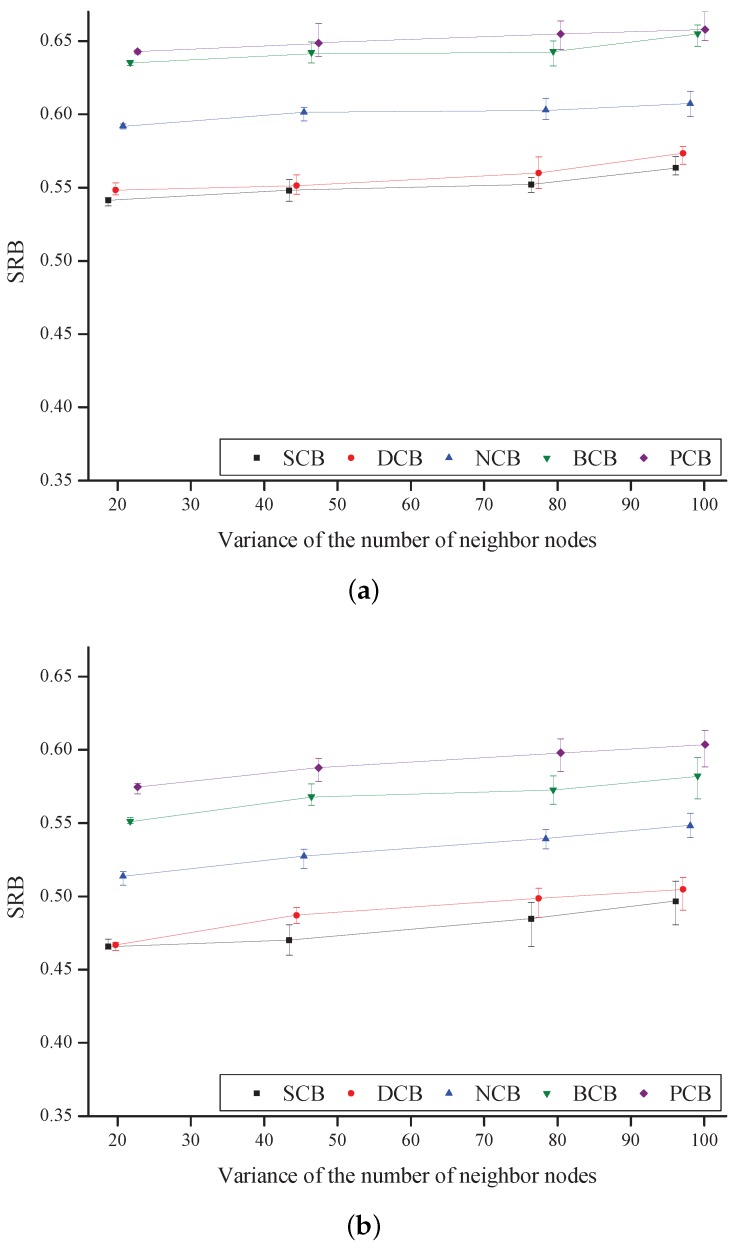

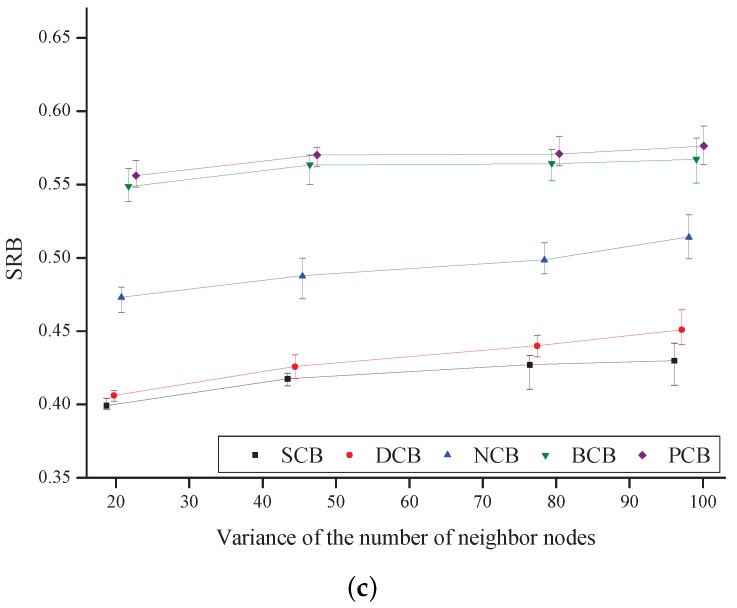


Figure 12 shows the SRB as a function of the variance in the number of neighbor nodes and of the count threshold value. For SCB, DCB, and NCB, $C_{max}$ is used as the count threshold value. In BCB and the proposed counter-based broadcast scheme, an adaptive count threshold is used, as shown in Equations (3) and (37). Generally, SRB increases as the variance of the number of neighbor nodes increases in all the schemes because SRB increases in high-node-density areas. We confirm that the proposed scheme outperforms the other four existing counter-based broadcast schemes in terms of SRB. This is because, under the proposed scheme, a node close to the broadcasting node or a node that cannot achieve a wider additional coverage area is more unlikely to retransmit the broadcast-message in a high-node-density region.

**Figure 13:**
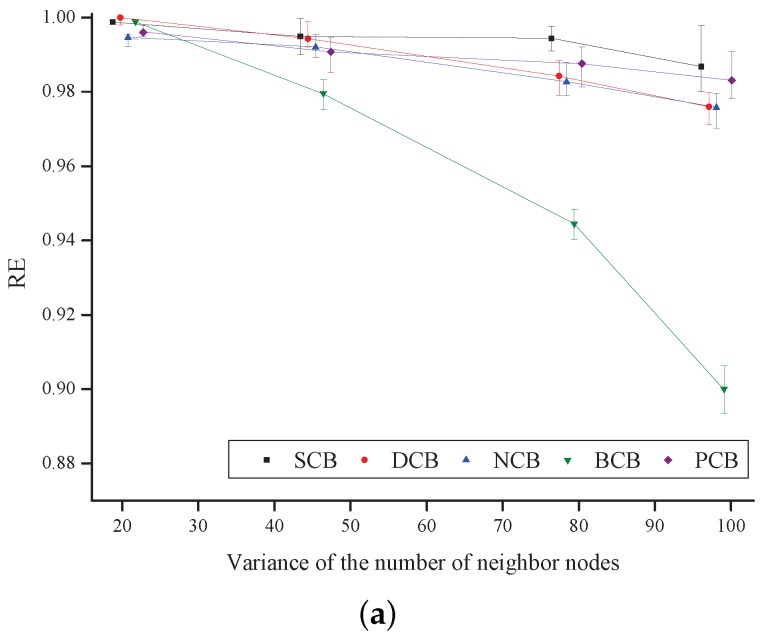

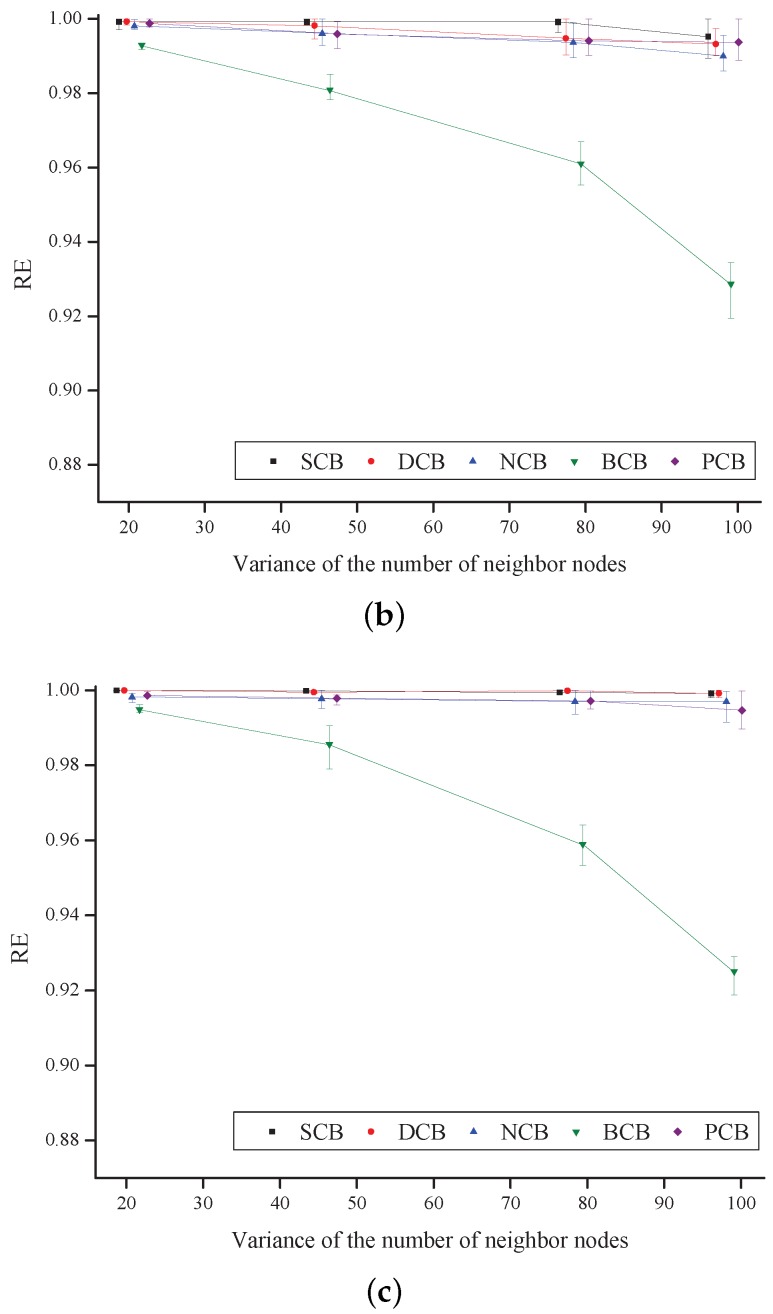


Figure 13 shows RE as a function of the variance of the number of neighbor nodes. Generally, RE decreases as the variance increases in all the schemes because disconnection occurs between the nodes located in the low-node-density regions. The simulation results show that BCB has the lowest reachability and SCB has the highest reachability. In addition, it is shown that the proposed scheme has reachability similar to or higher than that of the other existing schemes because, under the proposed scheme, a node that is far from the broadcasting node or a node that can achieve a wider additional coverage area is more likely to retransmit the broadcast-message.

**Figure 14:**
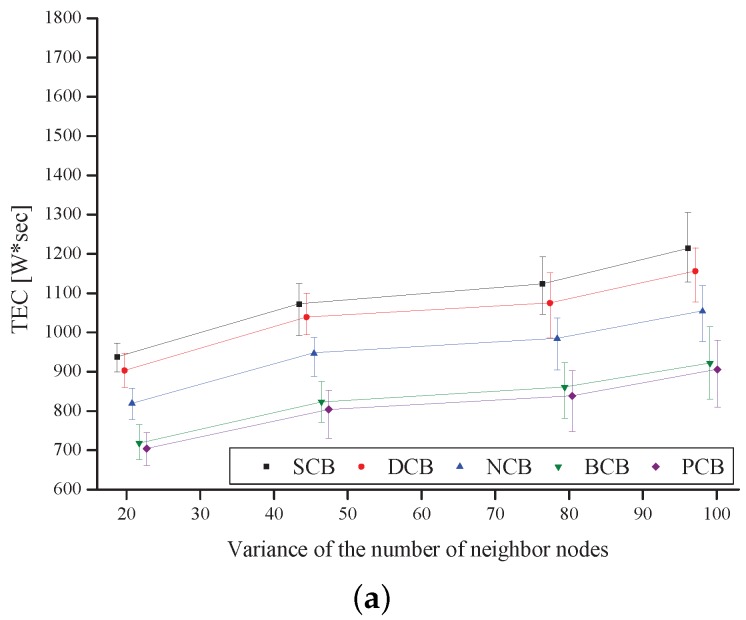

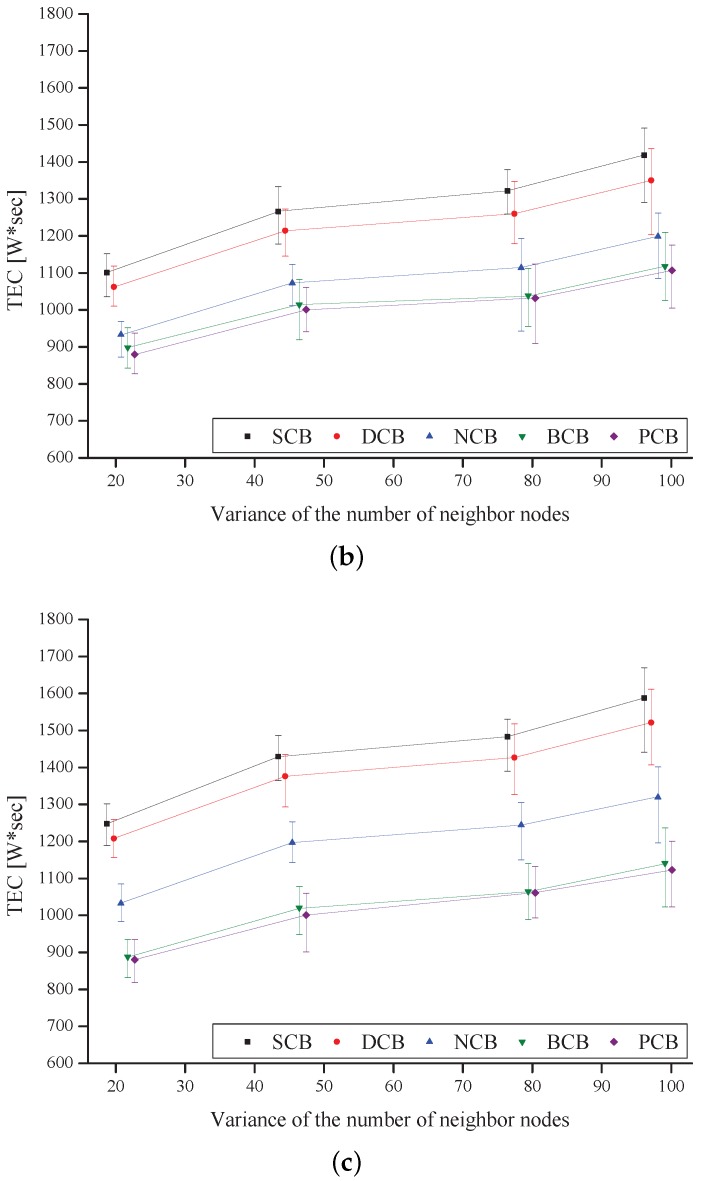


Figure 14 shows TEC as a function of the variance of the number of neighbor nodes. The result shows that TEC increases as $C_{max}$ increases, for all the schemes. In addition, TEC decreases as the variance increases in all of the schemes because the power consumptions of the nodes located in high-node-density regions decrease. It is noticed that the proposed scheme outperforms the other four existing counter-based broadcast schemes in terms of the TEC because a node that has a high remaining amount of battery retransmits the broadcast-message, under the proposed scheme.

## 5. Conclusions

In this paper, we proposed a counter-based broadcast scheme for WSNs, which was designed to consider the additional coverage area, distance, node density, and energy efficiency, simultaneously. First, we calculated the exact additional coverage area of a node ($AC_{k}$) to make the proposed scheme reflect the reachability accurately. RAD in the proposed scheme was composed of $RAD_{B}$, $RAD_{C}$, and $RAD_{D}$, which were determined by the remaining battery of a node $E_{k}$, the exact additional coverage area of a node $AC_{k}$, and the distance between a node and the broadcasting node $d_{tk}$, respectively. In addition, the weight factors for $RAD_{B}$, $RAD_{C}$, and $RAD_{D}$ were set according to the density of the node, so that a node in a dense area could achieve high energy efficiency and a node in sparse area could achieve both high reachability and low redundancy in transmitting the broadcast-message. On the other hand, the count threshold value $C_{th}$ was designed to be determined by either the remaining battery of a node or the network density. Specifically, the $C_{th}$ of a node in a sparse environment was set according to the number of neighbors to improve reachability, and that of a node in a dense environment was set by the remaining battery of the node to reduce the battery consumption of the node and redundancy of the broadcast-messages. Simulation results showed that the proposed counter-based broadcast scheme outperformed the previous SCB, DCB, NCB, and BCB schemes in terms of saved rebroadcasts, reachability, and total energy consumption. In future work, we have a plan to evaluate the performance of proposed broadcast scheme in a real testbed such as Twistlab, IoT Lab, or Indriya in order to verify the validity of the proposed broadcast scheme and confirm the possible constraints and efficiency issues.

## Figures and Tables

**Figure 6:**
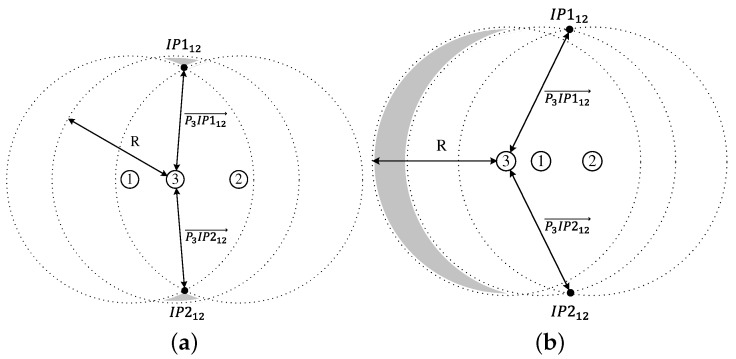

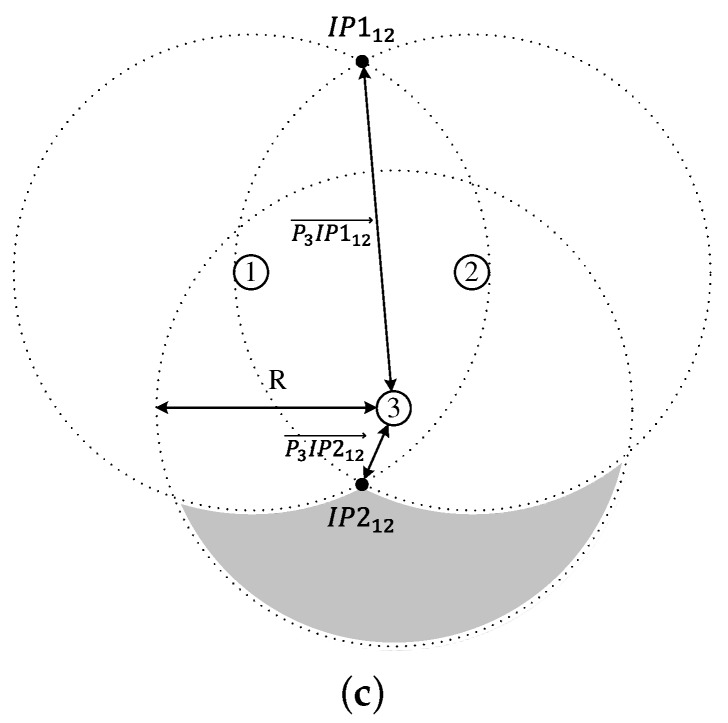


**Table 1:** Simulation parameters.

Parameter	Value
Topology	Ad-hoc network
Network size	500 × 500 m^2^
Number of nodes	120
Transmission range (R)	100 m
Packet type	Constant-bit-rate (CBR) packet
Packet generation period	1 s
Broadcast message length	200 bytes
Simulation run time	10 min
Energy distribution	Uniformly random in [80,100] W×s
Iteration	1000
$C_{min}$	1
$C_{max}$	3 ∼ 5
Variance of the number of neighbor nodes	20.73, 45.42, 78.41, 98.12
